# Metabolomics Reveals the Mechanism of Browning Inhibition by Transient Light Quality in Tea Plant Tissue Culture

**DOI:** 10.3390/plants14223539

**Published:** 2025-11-20

**Authors:** Yi Ding, Haitao Huang, Yun Zhao

**Affiliations:** Tea Research Institute, Hangzhou Academy of Agricultural Sciences, Hangzhou 310024, China; ydingone@163.com (Y.D.);

**Keywords:** light quality regulation, browning, polyphenols, metabolomics

## Abstract

The absence of a high-efficiency and stable genetic transformation system has been a critical bottleneck, impeding both functional gene characterization and precision breeding efforts in *Camellia sinensis* (tea), and browning is the first problem encountered in tissue culture of tea. In this paper, to identify optimal spectral conditions for minimizing browning in tissue culture, we subjected three tea plant cultivars to distinct light quality treatments and conducted comprehensive metabolomic profiling of their phytochemical contents. This study demonstrates that wavelength-specific light treatments can induce reversible modifications in the physicochemical characteristics of tea leaves, effectively reducing the accumulation of flavonoid compounds, including polyphenols, in plant tissues. Notably, tissues subjected to optimized wavelength conditions exhibit superior performance as explant sources for in vitro culture systems, demonstrating significantly lower browning rates. Comparative analysis of 460 nm, 660 nm, and 730 nm irradiation treatments revealed consistent suppression of polyphenol biosynthesis across all examined cultivars. However, the wavelength eliciting maximal reduction showed significant cultivar-dependent variation, indicating genotype-specific photoresponsive regulation of secondary metabolism.

## 1. Introduction

As one of the world’s three major beverages, *Camellia sinensis* (tea) serves as a vital economic pillar for many producing regions. Plant tissue culture has emerged as a pivotal technique with multifaceted applications spanning agricultural improvement, horticultural propagation, biological research, and conservation initiatives. This technology provides exact regulation over plant growth and genetic modification, leading to increased agricultural yields, improved phytosanitary control, and the protection of rare botanical species. However, the absence of a high-efficiency, stable genetic transformation system has been a critical bottleneck, impeding both functional gene characterization and precision breeding efforts in tea.

Browning is the first problem encountered in tissue culture of tea, which is a widely recognized constraint in plant tissue culture systems and presents significant obstacles to effective in vitro propagation. This phenomenon stems primarily from enzymatic oxidative processes triggered by explant injury. Uncontrolled browning can severely compromise tissue regeneration potential, suppress callus growth, inhibit adventitious shoot formation, and ultimately result in cellular necrosis [1]. Browning is very common in many plants such as *Camellia hainanica* Callus [2], *Isatis indigotica Fortune* [3], *Plantain* [4], and tea [5]. It has been found that this is mainly due to the accumulation of polyphenols.

To minimize the impact of browning in plant tissue culture, young tissues (such as shoot tips, embryonic buds, and newly emerged leaves) are often preferentially selected as explants during their developmental stage. Additionally, healthy plants free from pests and diseases with low lignification are prioritized [5,6,7]. To inhibit explant browning, various strategies have been developed, including presoaking explants in antioxidant solutions, incorporating antioxidants into the culture medium, maintaining cultures in darkness, and frequent sub-culturing [8]. Additionally, the encapsulation of natural products has emerged as a promising approach to mitigate browning in plant tissue culture [9]. Studies have shown that collecting explants in an antioxidant solution followed by washing with an antioxidant solution prior to inoculation can effectively reduce phenolic exudation [10]. Melatonin, as an antioxidant, is widely applied to prevent browning in broccoli [11]; anti-browning agents such as activated charcoal (AC) and polyvinylpyrrolidone (PVP) have been shown to improve the germination rate of seed embryos [12]. Addition of 225 mg/L citric acid + 50 mg/L ascorbic acid could alleviate phenol browning of Iranian Seedless Barberry [13]. The role of light in promoting phenolic exudation—an autocatalytic process—is well established, with studies confirming that cultures incubated in the dark typically exhibit reduced browning compared to those under light conditions [6,14,15]. Furthermore, exposure to light and elevated temperatures has been found to accelerate browning by enhancing enzymatic activity [16]. In *Plantain,* darkness reduces browning by suppressing decay and decreasing the activity of enzymes associated with phenolic compounds [4]. Similarly, in *Saurauia bracteosa*, combining activated charcoal with dark incubation delayed browning onset, while the use of ascorbic acid under dark conditions resulted in the lowest browning intensity [17]. Low-temperature dark treatment has also proven effective: in *J. mandshurica*, storing budded stems at low temperature in darkness for five days inhibited browning and promoted bud sprouting [18], and similarly, in *Paeonia ostii*, dark incubation at 4 °C enhanced in vitro propagation while effectively suppressing browning [19]. Studies have indicated that the forcing treatment can induce a significant decrease in endogenous total phenolic levels of explants prior to cultivation, and this reduction is closely correlated with mitigated explant browning severity as well as enhanced survival rates in grapevine [20].

Light serves as a critical environmental regulator that influences plant growth and development through dual roles: as a fundamental energy source for photosynthesis and as a key signaling stimulus. Research has established light as a critical environmental factor influencing tea plant growth and quality [21,22]. It drives flavonoid biosynthesis, which serves a protective function under high-light stress [23]. Studies indicate that high light intensity promotes the accumulation of flavonoids, phenolics, and anthocyanins [24], in contrast to shading, which reduces them [25]. In contrast to its effect on flavonoids, shading has been demonstrated as an effective practice for boosting theanine content [26,27]. Additionally, both catechin levels and photosynthetic capacity respond non-linearly to light, increasing under moderate intensity but decreasing under extremes of low or high light [28,29,30]. The influence of light on plant tissue culture is primarily manifested in three aspects: light quality, light intensity, and photoperiod. For instance, a study on *Cunninghamia lanceolata* tissue culture seedlings demonstrated that a specific light quality ratio of red/blue/purple/green at 8:1:1:1 resulted in a superior rooting rate and enhanced root growth [31]. In the case of *Tilia miqueliana* callus, the optimal protocol involved an initial 10-day dark culture, followed by sequential exposure to low light intensity (500 lx) for 5 to 7 days and then transfer to a higher intensity of 2000 lx [32]. However, effective strategies to control browning in tea plant tissue culture through light regulation remain limited. This study therefore aims to identify optimal spectral conditions for minimizing browning in tissue culture. We subjected three tea plant cultivars to distinct light quality treatments and conducted comprehensive metabolomic profiling of their phytochemical contents.

## 2. Results

### 2.1. Leaf Morphology Identification of LJ43 Which Exposed to Irradiations of 3 Different Light

**Figure 1:**
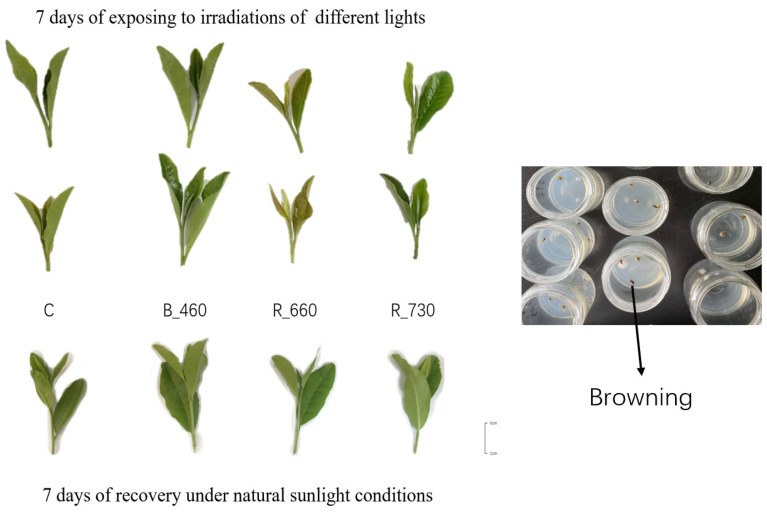
Leaf morphology identification of LJ43, which were exposed to the irradiations of three different lights and recover under sunlight (**left**). Browning of in vitro explants (**right**).

The tea plants cultivar LJ43 were exposed to irradiations of blue light (460 nm), red light (660 nm), near-infrared light (730 nm), and sunlight (control) for 7/17 h light/dark for 7 days. The experimental results demonstrated that the foliar morphology of tea shoots at the one-bud-two-leaf stage exhibited significant alterations following light treatments compared to the control group. Notably, distinct phenotypic variations were observed among different wavelength treatments: (1) under 460 nm illumination, treated leaves displayed a darker green pigmentation accompanied by increased thickness of both buds and leaves; (2) exposure to 660 nm light induced pronounced reddish leaf coloration along with reduced organ thickness; (3) the 730 nm treatment group showed minimal morphological changes. Importantly, these light-induced phenotypic modifications were transient, as all treated samples reverted to normal morphological characteristics comparable to control plants following one week of recovery under natural sunlight conditions (Figure 1).

### 2.2. Amino Acid (AA), Polyphenols (PP) and Phenol-Ammonia Ratio (PP/AA) Contents Identification of LJ43 Exposed Under 3 Different Lights

**Figure 2:**
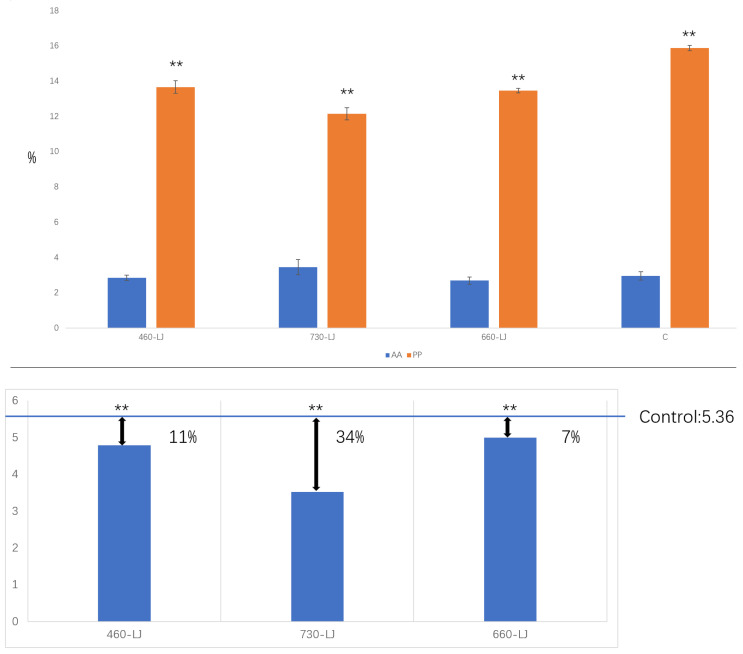
AA, PP (**up**), and PP/AA (**down**) contents of LJ43, which were exposed to the irradiations of three different lights (C: control, **: *p* < 0.01).

In addition to leaf morphology, there were significant differences in the nutrient metabolite contents of AA, PP, and PP/AA. The experimental data revealed that the PP/AA in LJ43 exhibited a significant reduction across all three light wavelength treatment groups compared to the control. Notably, the most pronounced decrease (34%) was observed in the 730 nm treatment group. Subsequent analytical findings indicated that this alteration was primarily attributable to a reduction in PP content, with all three experimental treatment groups exhibiting significantly lower PP levels compared to the control group (Figure 2).

### 2.3. Tissue Culture and Browning Rate Statistics

Axillary buds of LJ43 obtained from both the control group and experimental groups treated with three different lights were cultured in vitro. Following a 7-day incubation period, contamination and browning rates were assessed and subjected to statistical analysis. The results showed that there was no significant change in the contamination rate of tissue culture, but the browning rate was significantly reduced in the treatment group, especially in the 730 nm treatment group, where it decreased from 11.3% to 2% (Table 1).

**Table 1:** Browning rates of LJ43 which exposed to irradiations of 3 different light and control.

	Browning Rate
Control	11.3% a
660 nm group	8.0% b
730 nm group	2.0% c
460 nm group	7.0% b

Different letters after the values refer to significant difference at the *p* < 0.01.

### 2.4. Untargeted Metabolomics Contents Identification of LJ43 Exposed Under 3 Different Lights

**Figure 3:**
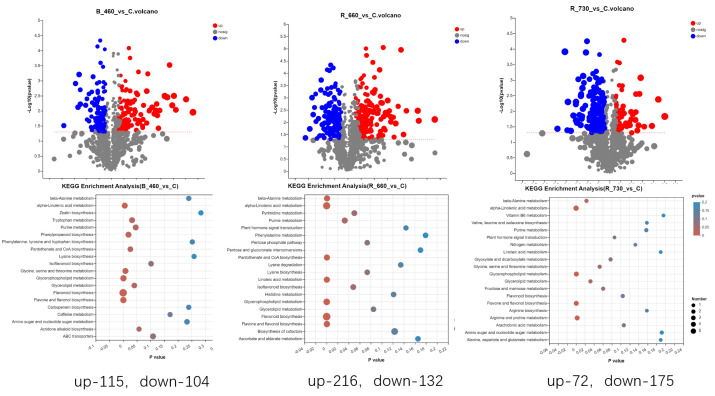
Upregulated metabolites and downregulated metabolites of LJ43, which were exposed to the irradiations of three different lights (**up**); KEGG metabolic pathways of LJ43, which were exposed to the irradiations of three different lights (**down**).

Through research, it was found that in the 460 nm treatment group, compared to the control group, LJ43 had 115 upregulated metabolites and 104 downregulated metabolites; in the 660 nm treatment group, 216 metabolites were upregulated and 132 were downregulated; in the 720 nm treatment group, only 72 metabolites were upregulated and 175 were downregulated; and the KEGG metabolic pathways enriched by these metabolites were similar, such as porphyrin and chlorophyll metabolism, flavone and flavonol biosynthesis alpha linolenic acid metabolism and flavonoid biosynthesis (Figure 3).

**Figure 4:**
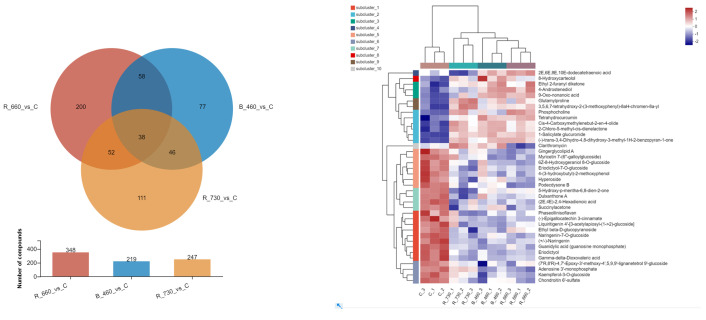
Differential metabolites of LJ43, which were exposed to the irradiations of three different lights ((**Left**)-Number of differential metabolites and co-occurrence under different wavelength treatments; (**Right**)-Expression levels of 38 metabolites showing differences across all three wavelengths treatments).

An intergroup comparative analysis of the metabolic alterations demonstrated that the 660 nm wavelength treatment group exhibited the highest number of differentially expressed metabolites, whereas the 460 nm wavelength treatment group displayed the lowest quantity. Notably, among these differentially regulated metabolites, 38 compounds manifested statistically significant variations across all three experimental groups, suggesting their potential susceptibility to photic stimulation (Figure 4 left). Intergroup comparative analysis of these 38 metabolites revealed that their changes were generally consistent compared with the control. Among the three treatment groups, 730 showed different trends in three metabolites compared to the other two treatment groups, including 4-Androstenediol, etc. (Figure 4 right).

**Figure 5:**
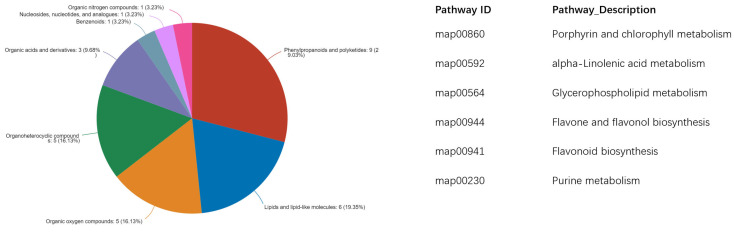
Classified compounds (**left**) and involved pathways (**right**) of 38 metabolites showing differences across all three wavelengths.

Among these 38 metabolites, 9 were classified as phenylpropanoids and polyketides, 6 as lipids and lipid-like molecules, 5 as organic oxygen compounds, 5 as organoheterocyclic compounds, and 5 as organic acids and derivatives. The pathways involving these metabolites included porphyrin and chlorophyll metabolism, alpha-Linolenic acid metabolism, glycerophospholipid metabolism, flavone and flavonol biosynthesis, flavonoid biosynthesis, and purine metabolism (Figure 5).

**Figure 6:**
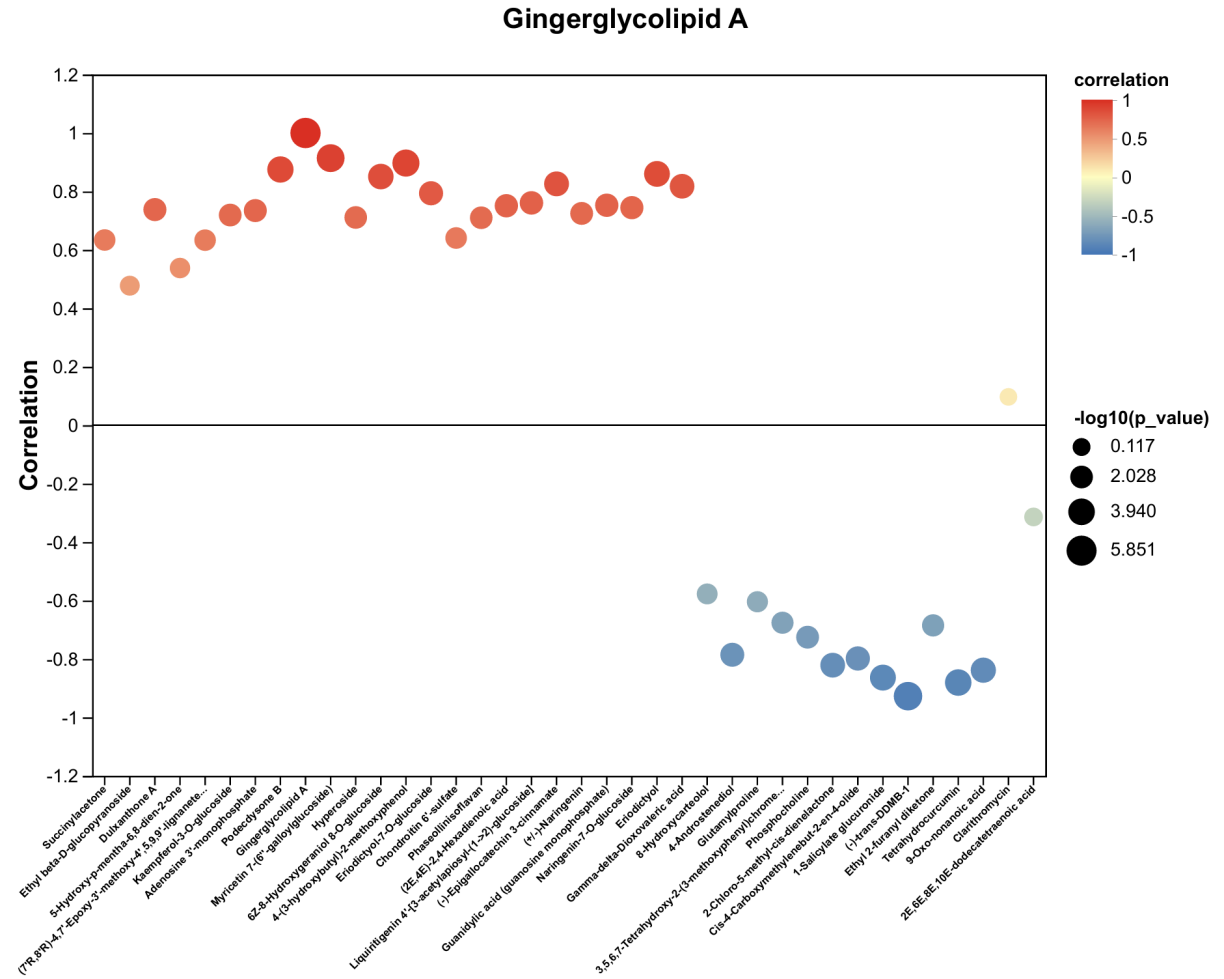

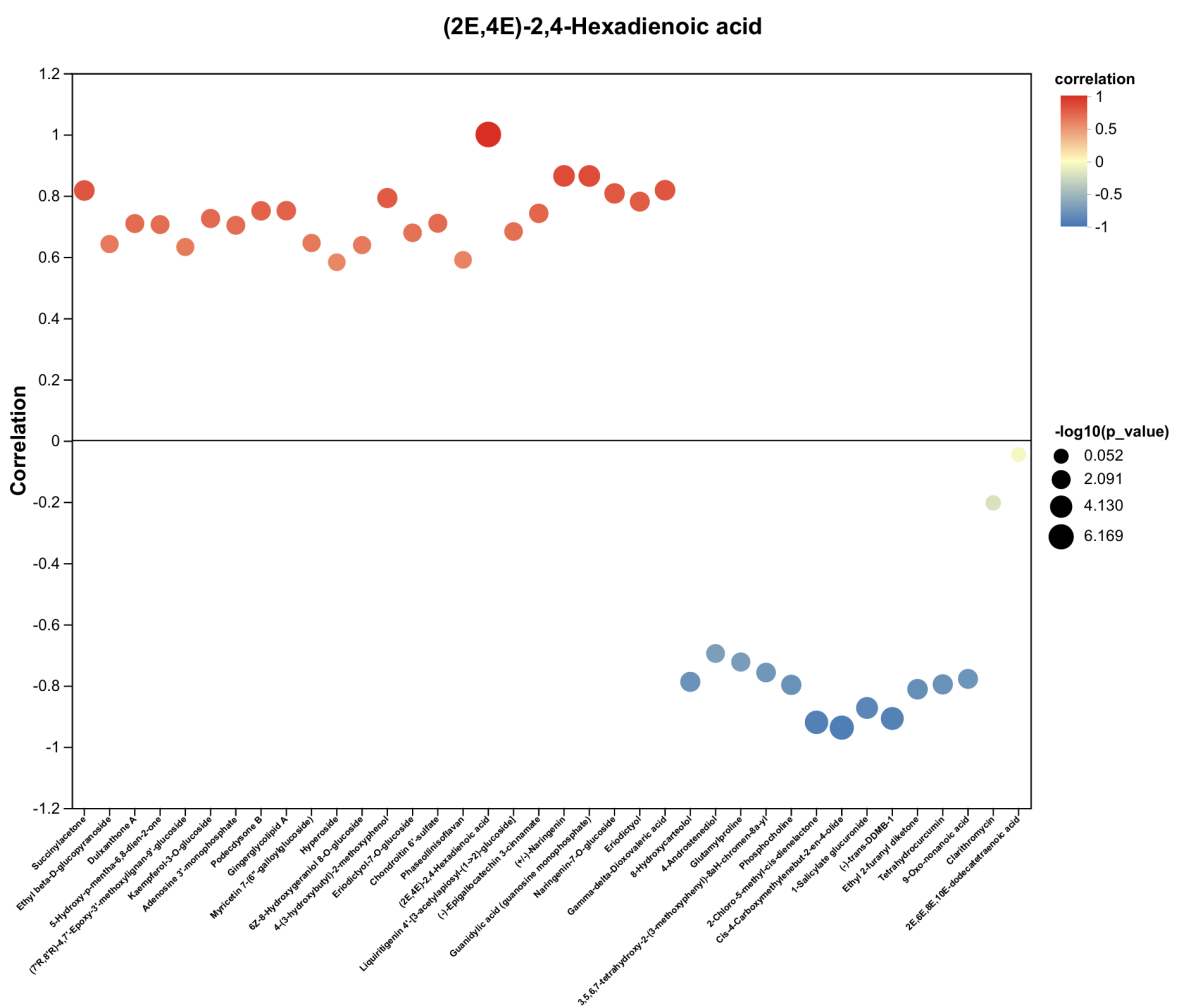

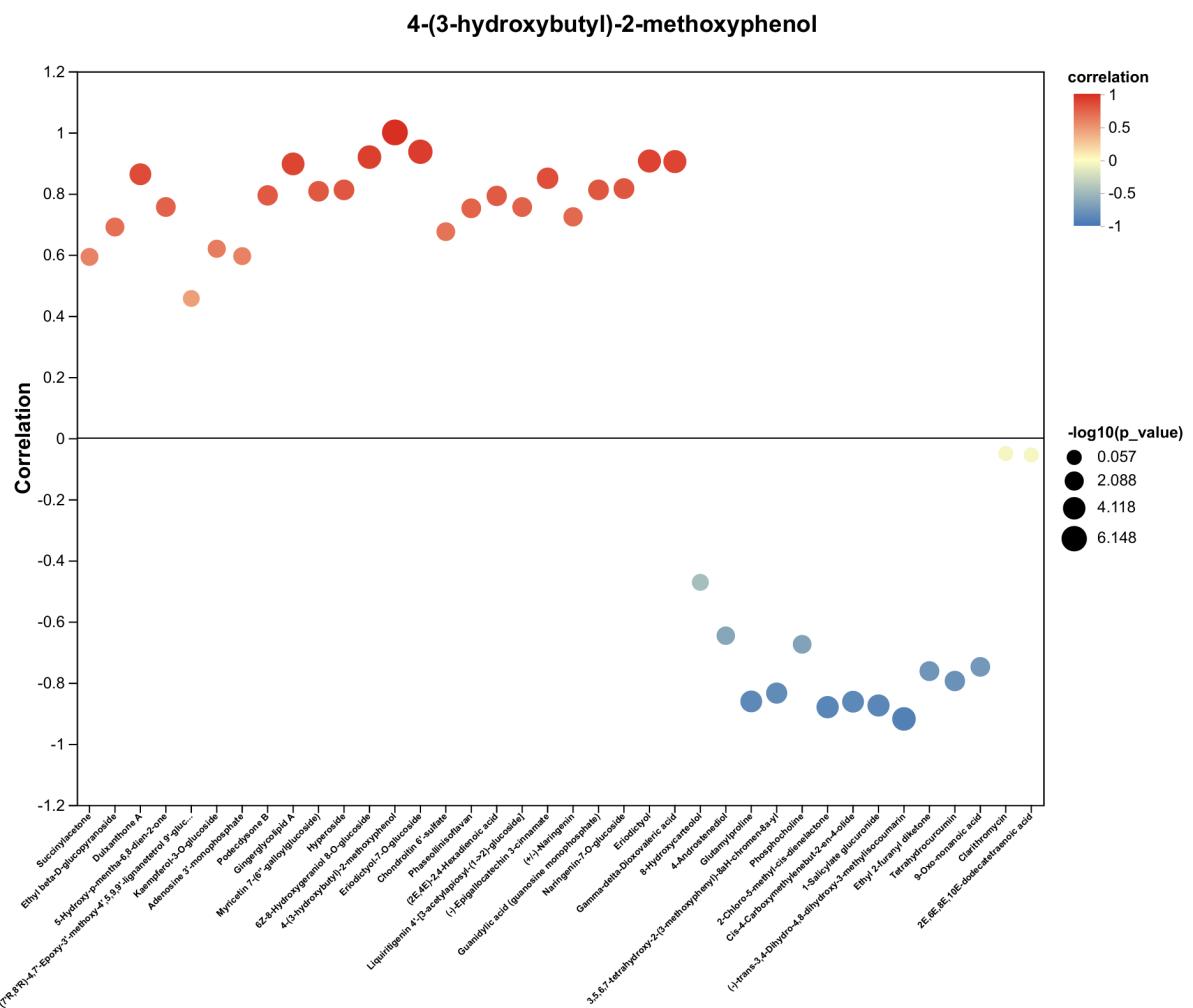

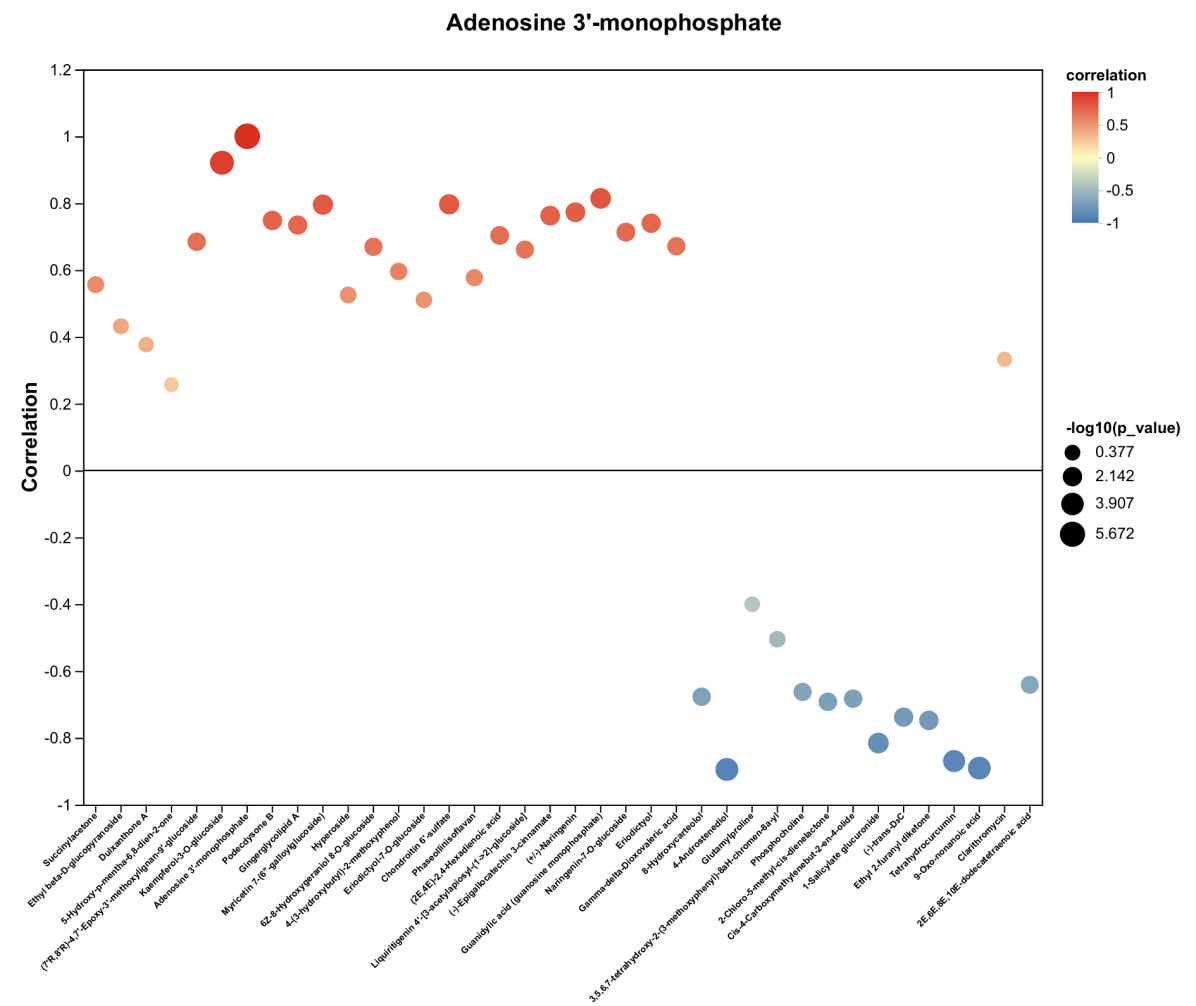

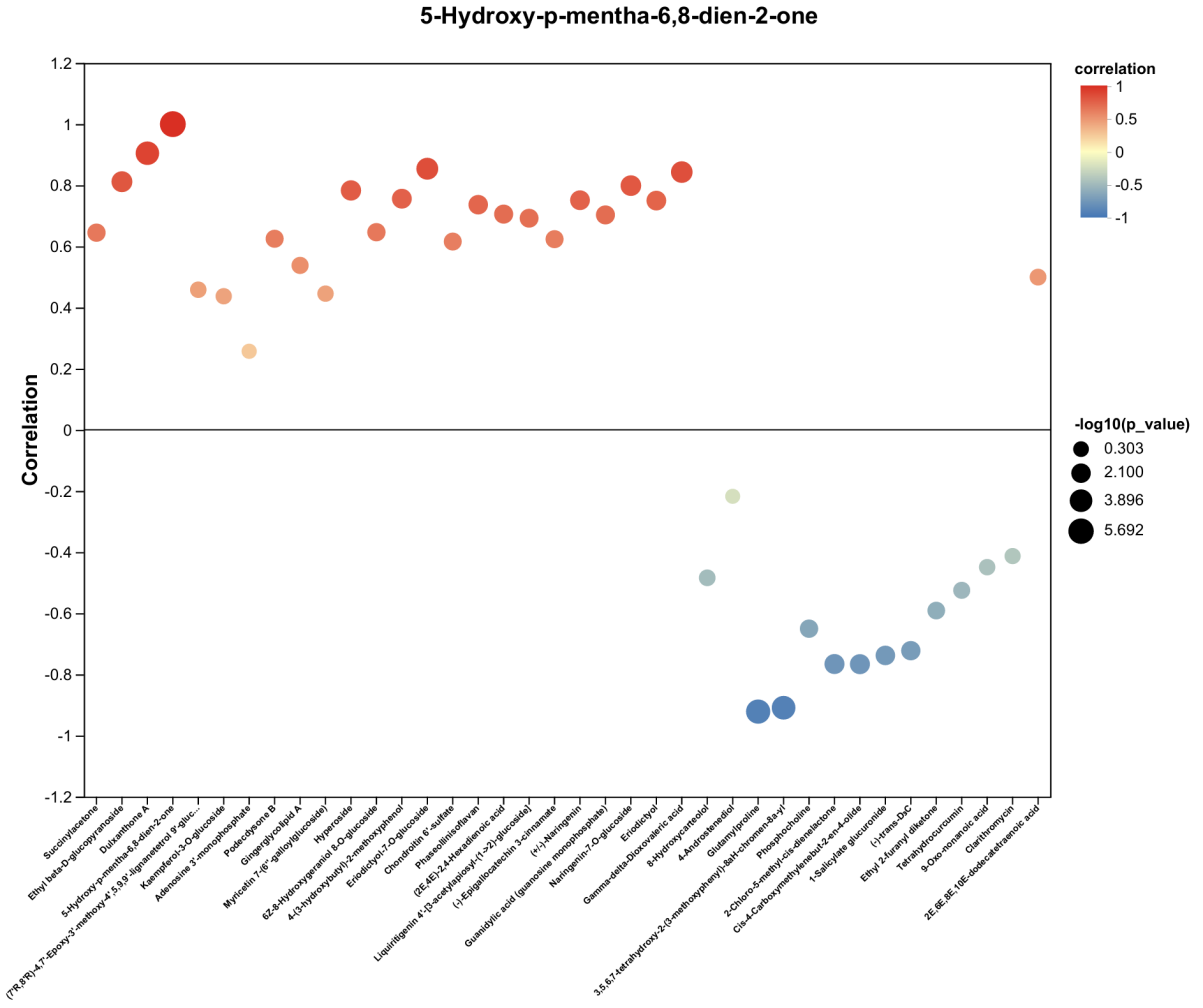

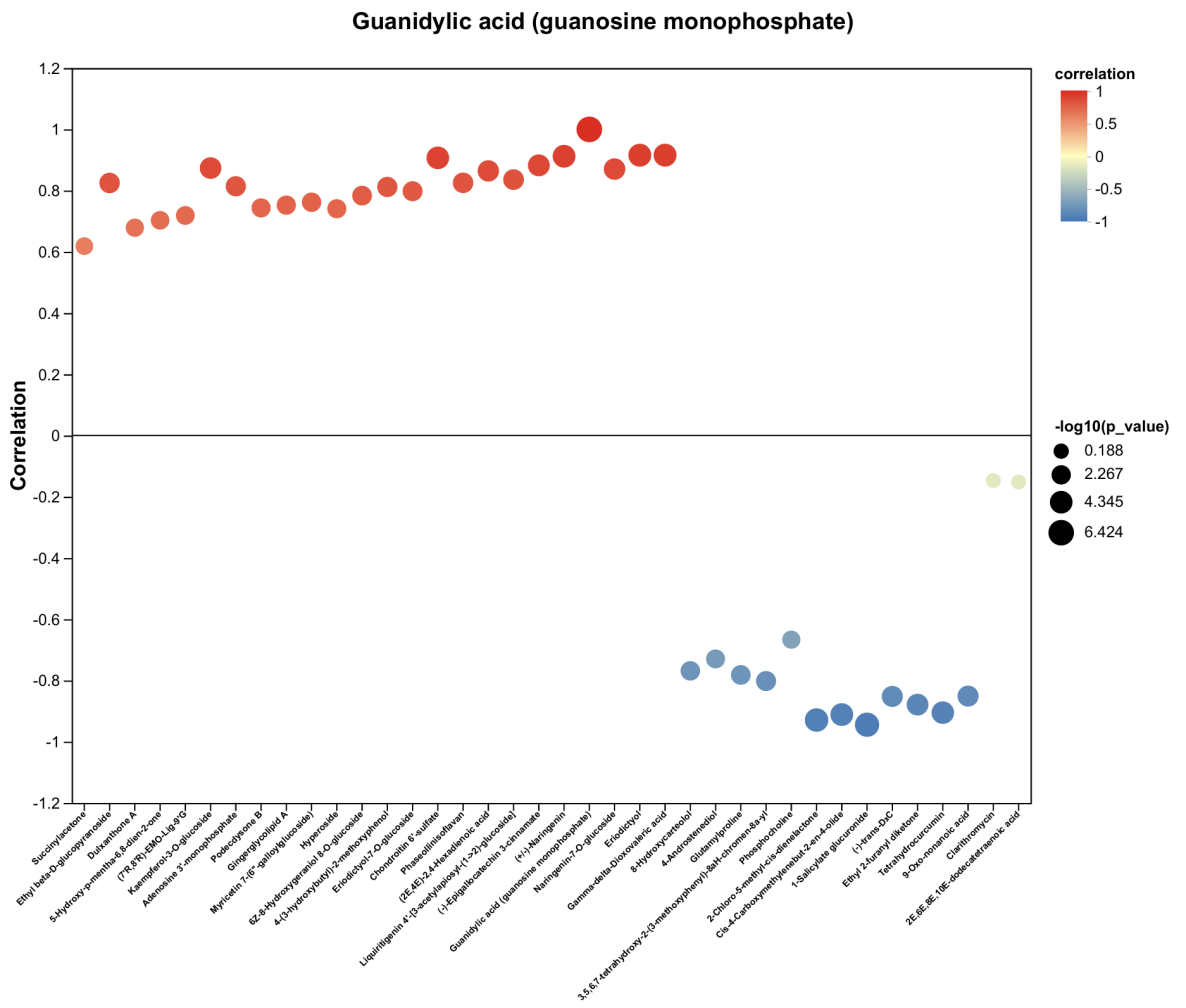

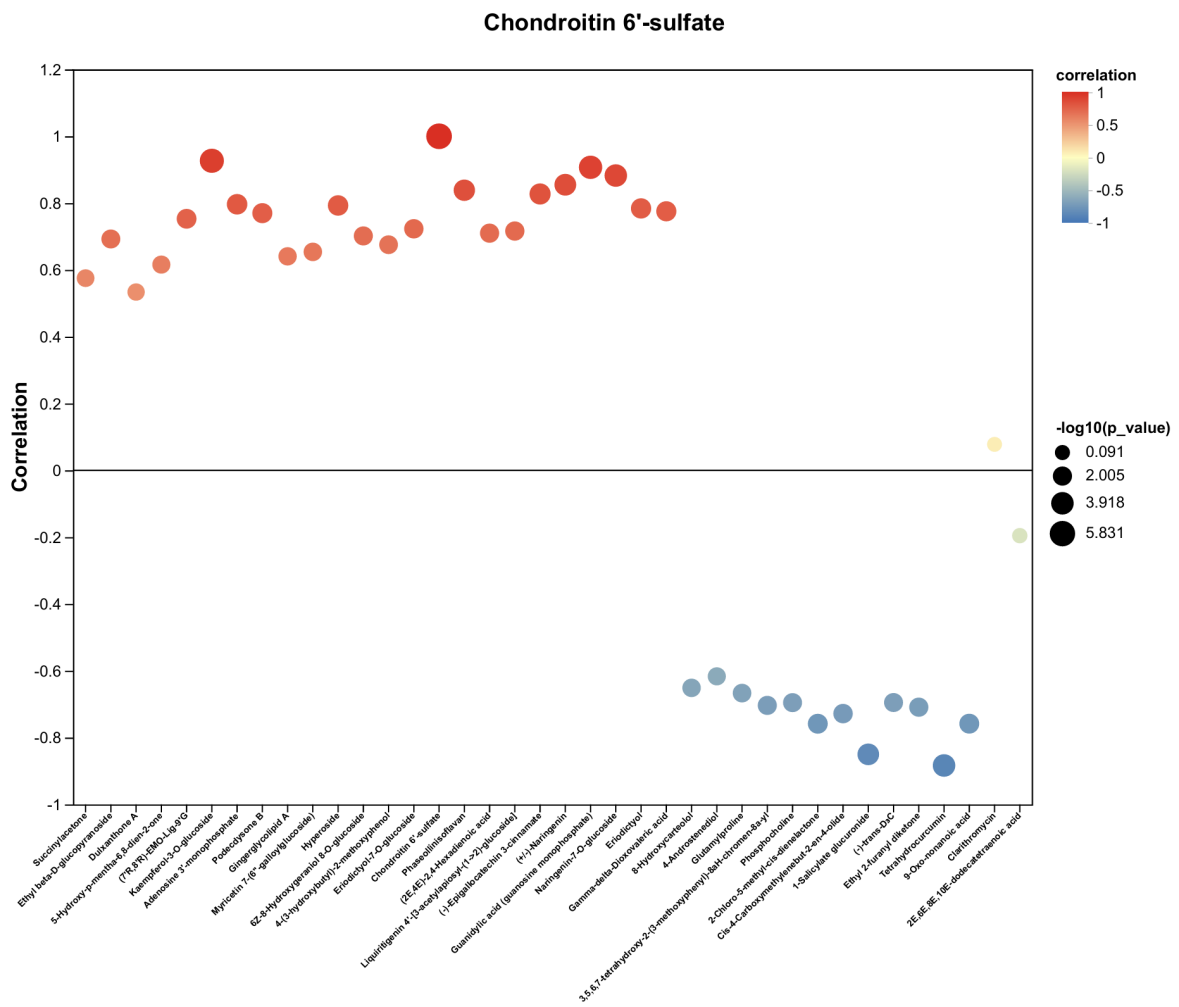

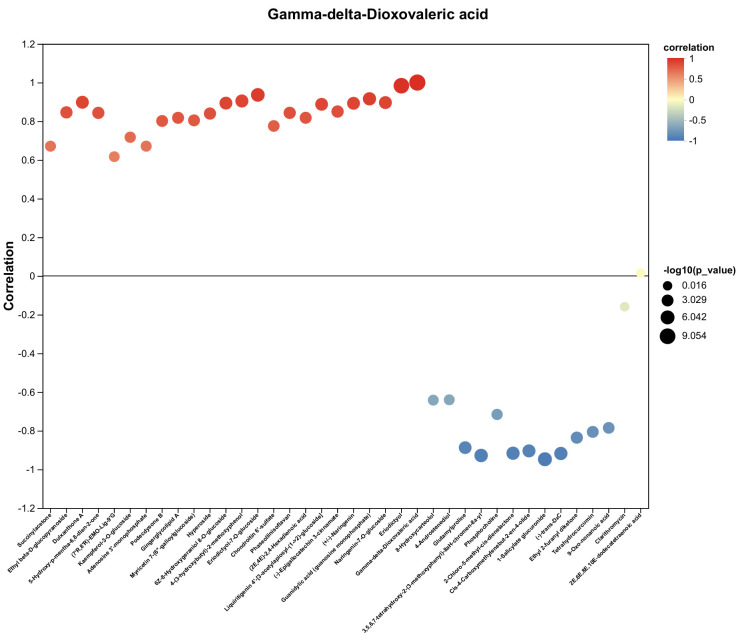

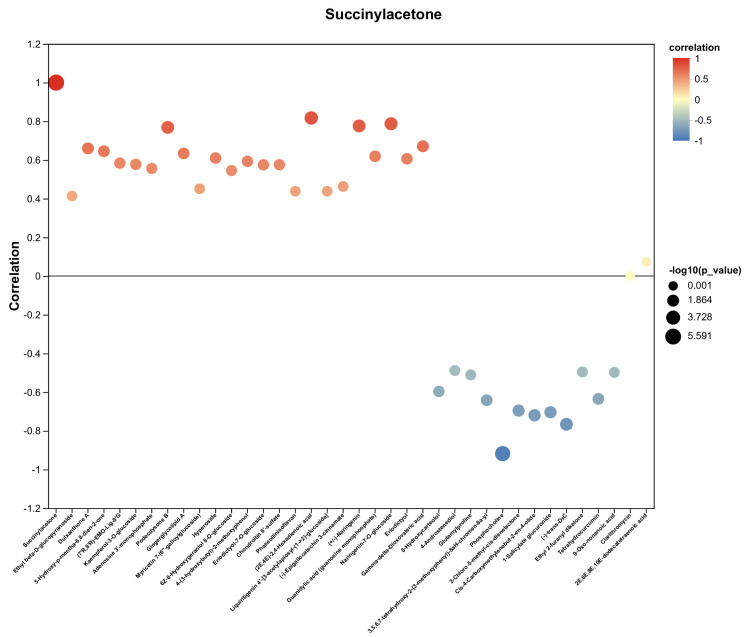

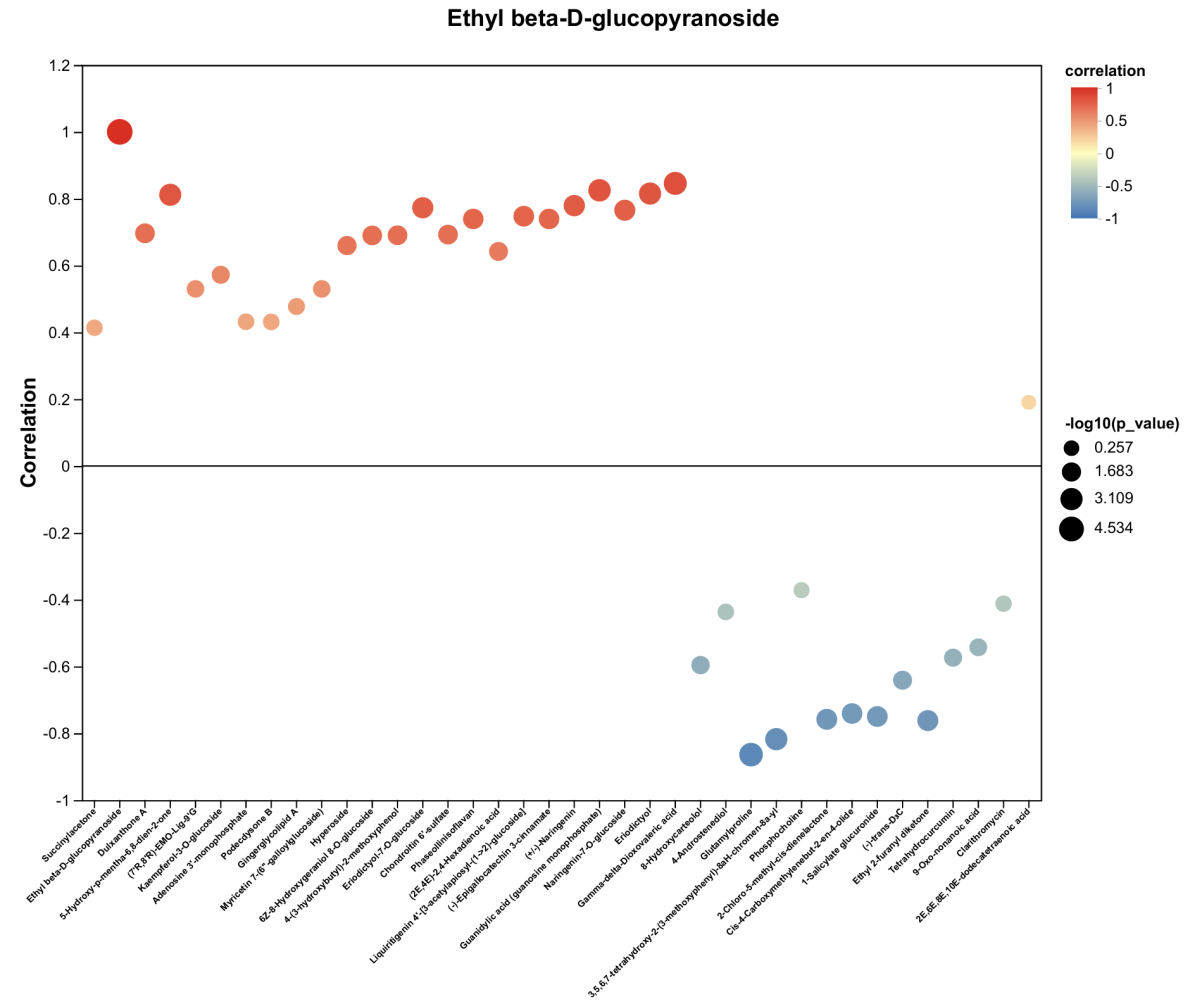
Ten non-flavonoid metabolites exhibited significant positive correlations with flavonoid compounds.

Among these 38 metabolites, 24 exhibited a significant decrease relative to the control group (Table 2). Of these, 14 were identified as flavonoids, like (-)-Epigallocatechin 3-cinnamate, while the remaining 10 non-flavonoid metabolites exhibited significant positive correlations with flavonoid compounds (Figure 6). Specifically, gingerglycolipid A demonstrated a positive correlation with myricetin 7-(6′-galloylglucoside), while (2E,4E)-2,4-hexadienoic acid showed correlation with podecdysone B. Similarly, adenosine 3′-monophosphate was positively associated with kaempferol-3-O-glucoside, and 5-hydroxy-p-mentha-6,8-dien-2-one correlated with dulxanthone A. Furthermore, chondroitin 6′-sulfate displayed positive correlation with kaempferol-3-O-glucoside, and γ-δ-dioxovaleric acid was associated with both eriodictyol and dulxanthone A. Additionally, ethyl β-D-glucopyranoside and guanidylic acid (guanosine monophosphate) were found to correlate positively with dulxanthone A.

### 2.5. Untargeted Metabolomics Contents Identification of Other Tea Cultivars Exposed Under 3 Different Lights

**Figure 7:**
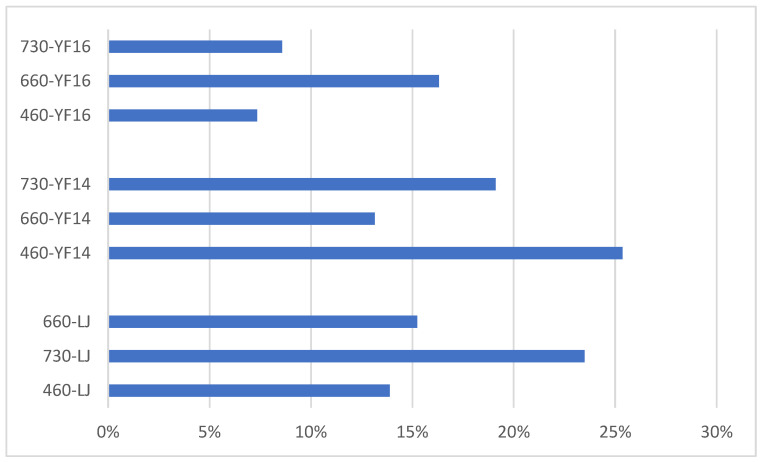
The proportion of tea polyphenols decreased under different light treatments for different cultivars.

Identical light treatments were applied to additional tea cultivars. In YF14 and YF16, the tea PP content demonstrated a significant decline in the treated groups compared to the control. The extent of PP reduction varied across cultivars depending on the light wavelength. Notably, LJ43 exhibited the most pronounced decrease in PP levels under 730 nm irradiation, whereas YF16 showed the greatest reduction under 660 nm light. In contrast, YF14 displayed the largest decline following 460 nm light treatment (Figure 7).

**Figure 8:**
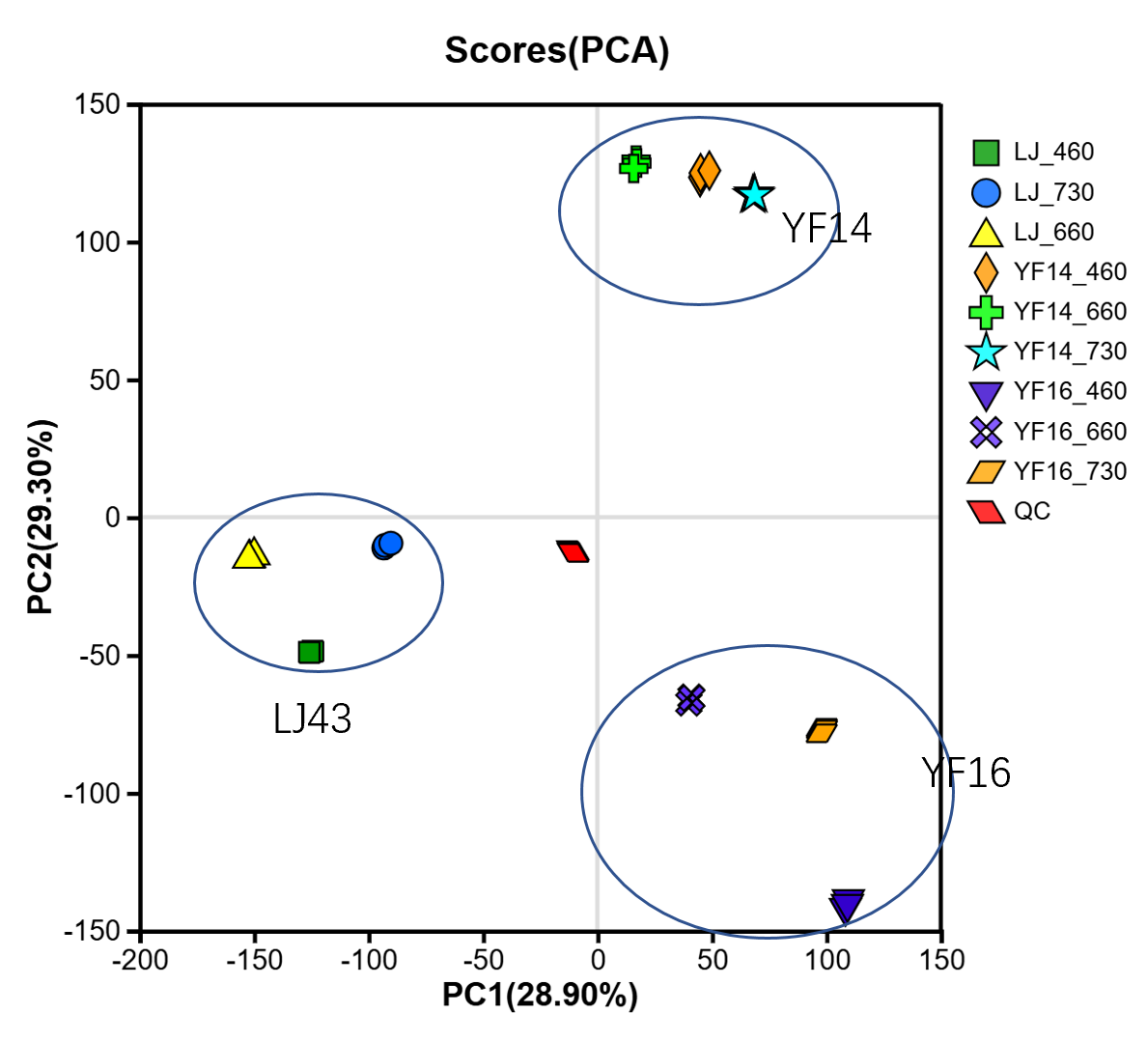
Principal component analysis (PCA) of metabolomic profiles from three tea cultivars (LJ43, YF14, and YF16).

Principal component analysis (PCA) of metabolomic profiles from three tea cultivars (LJ43, YF14, and YF16) subjected to three distinct light wavelengths revealed that genetic variation exerts a significantly stronger influence on metabolite composition than photoperiod treatment conditions. Furthermore, the metabolic signatures of different cultivars were clearly distinguishable in multivariate space (Figure 8).

**Figure 9:**
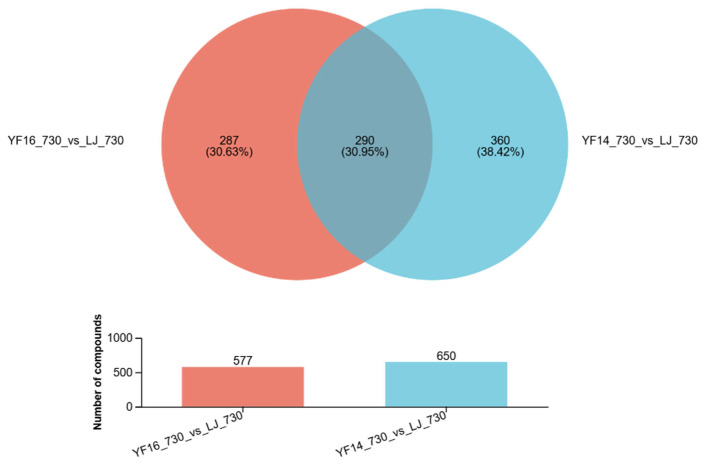
Comparative metabolomic analysis revealed substantial differences among cultivars.

Given that the 730 nm treatment group exhibited the most pronounced reduction in both PP content and PP/AA among all experimental groups in LJ43, we subsequently conducted a comparative analysis of YF14 and YF16 performance specifically under the 730 nm irradiation condition. Metabolomic profiling revealed significant inter-varietal differences in metabolite composition, with cultivar YF14 demonstrating a greater number of differentially expressed metabolites relative to LJ43 compared to YF16. As Figure 9 showed, comparative metabolomic analysis revealed substantial differences among the cultivars, with 287 unique differential metabolites identified between YF16 and LJ43, and 360 between YF14 and LJ43. Notably, 290 differential metabolites were common to both YF16 and YF14 when compared to LJ43.

**Figure 10:**
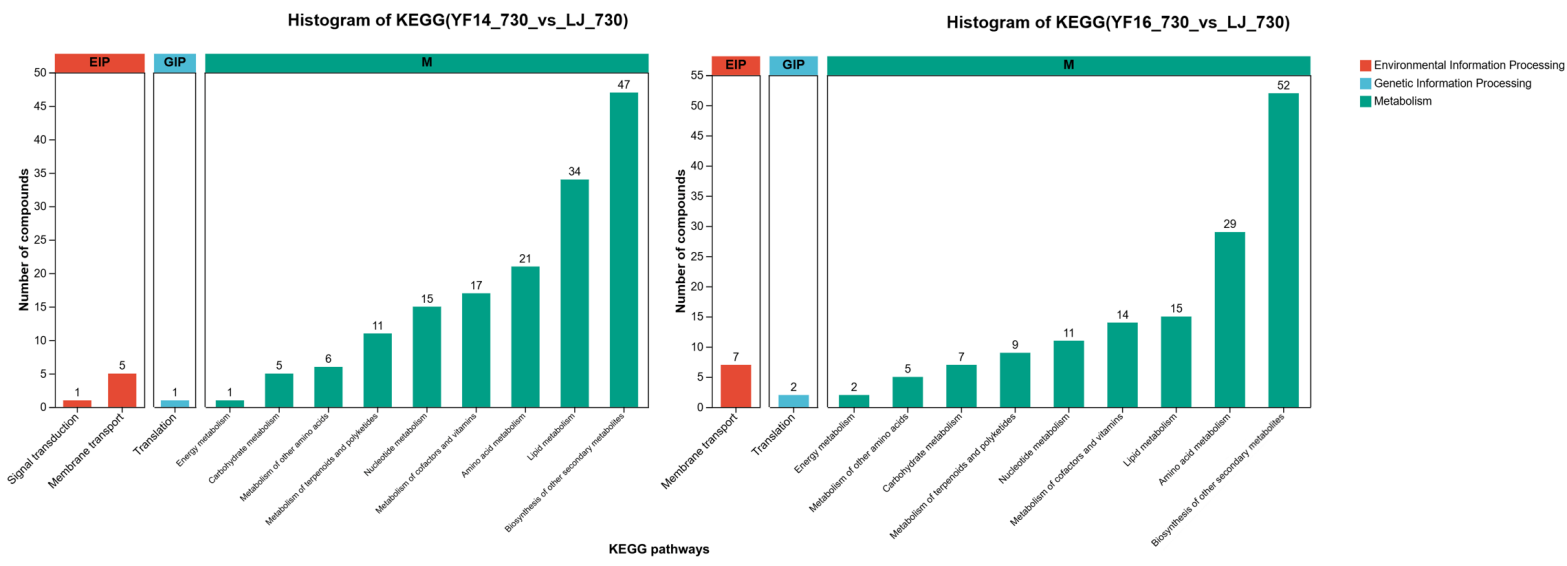
KEGG analysis of differentially expressed metabolites among different cultivars.

KEGG analysis of these differentially expressed metabolites revealed that, regardless of whether in YF14 or YF16, the majority of differentially expressed metabolites were concentrated in metabolic pathways, specifically distributed in the biosynthesis of other secondary metabolites, lipid metabolism, and amino acid metabolism pathways (Figure 10).

**Figure 11:**
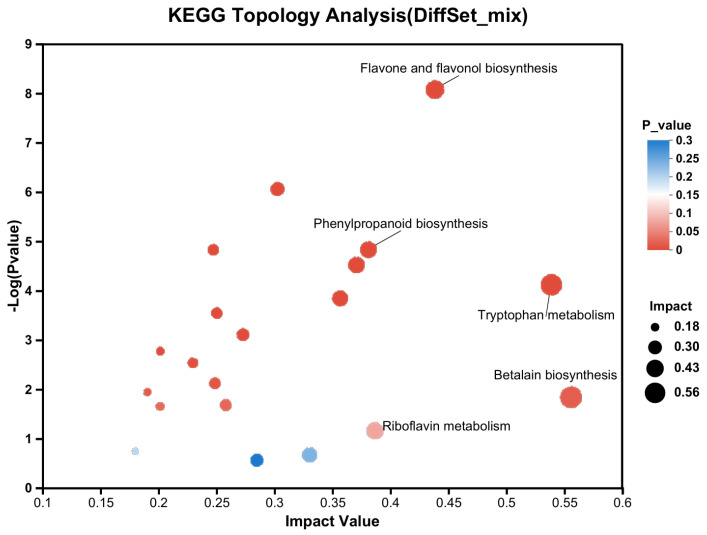
KEGG of differentially expressed metabolites from three tea cultivars under three distinct light wavelengths. The *x*-axis represents the impact value of metabolites within the pathways, indicating their relative importance. The *y*-axis shows the enrichment significance of metabolites involved in the pathways, expressed as −log10 (*p*-value). The bubble size corresponds to the impact value.

Metabolomic analysis of the three tea cultivars under three distinct light wavelengths revealed significant inter-varietal differences in metabolic profiles. As illustrated in Figure 11, these differential metabolites were predominantly enriched in several key biosynthetic pathways, including flavone and flavonol biosynthesis, phenylpropanoid biosynthesis, tryptophan metabolism, and betalain biosynthesis.

## 3. Discussion

### 3.1. Optimal Tissue Culture Materials for Reducing Browning Rates

The morphology of leaves subjected to different wavelengths of light exhibits discernible alterations, with the most prominent being variations in leaf coloration. These morphological changes differ significantly across wavelength-specific treatment groups. Notably, the observed modifications are reversible; upon subsequent exposure to natural light conditions for a defined duration (7 days), the leaf morphology reverts to a state indistinguishable from that of the control group. This suggests that the physicochemical changes induced by wavelength-specific light treatments are transient in nature. Short-term light exposure may only elicit temporary morphological adaptations without impairing the intrinsic growth potential of leaf cells. This finding supports the feasibility of utilizing axillary buds derived from wavelength-treated leaves as viable explants for tissue culture, as their cellular proliferative capacity remains unaltered.

In addition, the experimental data revealed that the PP/AA in LJ43 exhibited a significant reduction across all three light wavelength treatment groups compared to the control. Notably, the most pronounced decrease (34%) was observed in the 730 nm treatment group. Subsequent analytical findings indicated that this alteration was primarily attributable to a reduction in PP content. Axillary buds of LJ43 obtained from both the control group and the experimental groups treated with three different lights were cultured in vitro. The results showed there was no significant change in the contamination rate of tissue culture, but the browning rate was significantly reduced in the treatment group, especially in the 730 nm treatment group, where it decreased from 11.3% to 2%.

Previous studies have pointed out that browning is very common in many plants, and the browning rate is often proportional to the polyphenolic content [2,3,4,5]. These findings are consistent with our experimental results, confirming that explants with lower polyphenol (PP) content exhibit reduced browning incidence. To suppress browning, various strategies have been developed, ranging from presoaking explants in antioxidant solutions and incorporating antioxidants into the culture medium to maintaining cultures in darkness and implementing frequent sub-culturing [6]. These methods rely on the addition of exogenous compounds and do not alter the polyphenol content of the explants themselves, which may potentially affect the success of subsequent steps. In our experiment, we employed a simple method of supplementing with three different wavelengths of exogenous light. Compared to the control group, the LJ43 cultivar exhibited a decrease in tea polyphenols, and these differential changes disappeared after the various light treatments were withdrawn for a period of time. Furthermore, reversible light treatment represents a viable strategy for generating tissue culture materials characterized by both low tea polyphenol accumulation and suppressed browning rates.

### 3.2. The Mechanism of Transient Light Quality Modulation on Browning Inhibition in Tea Plant Tissue Cultural

Further analysis of the treatment group through Untargeted Metabolomics Contents Identification of LJ43 exposed under three different lights revealed that in the 460 nm treatment group, compared to the control group, LJ43 had 115 upregulated metabolites and 104 downregulated metabolites; in the 660 nm treatment group, 216 metabolites were upregulated and 132 were downregulated; in the 720 nm treatment group, only 72 metabolites were upregulated and 175 were downregulated; and the KEGG metabolic pathways enriched by these metabolites were similar, such as porphyrin and chlorophyll metabolism, flavone and flavonol biosynthesis alpha linolenic acid metabolism and flavonoid biosynthesis. It is explained that treatment with different wavelengths of light has a significant effect on photosynthetic pathways and secondary metabolites such as flavonoids.

Among these differentially regulated metabolites, 38 compounds manifested statistically significant variations across all three experimental groups, and 9 were classified as phenylpropanoids and polyketides, 6 as lipids and lipid-like molecules, 5 as organic oxygen compounds, 5 as organoheterocyclic compounds, and 5 as organic acids and derivatives. In addition, among these 38 metabolites, 24 exhibited a significant decrease relative to the control group, and 14 were identified as flavonoids, like (−)-Epigallocatechin 3-cinnamate, while the remaining 10 non-flavonoid metabolites exhibited significant positive correlations with flavonoid compounds. These results suggest that flavonoids, functioning as secondary metabolites, exhibit inducibility to variations in light wavelength conditions. As a key class of flavonoid compounds in tea, PP demonstrate wavelength-dependent regulation similar to other flavonoids. This photoregulatory mechanism enables the selection of plant material exhibiting reduced PP expression levels, which proves advantageous for explant preparation in tissue culture applications. In the LJ43 tea cultivar, exposure to 730 nm wavelength irradiation resulted in minimal PP accumulation and significantly reduced browning incidence, suggesting this photoperiodic treatment represents the optimal condition for explant establishment in tissue culture systems.

### 3.3. Different Wavelength Light Treatment Conditions on Different Tea Cultivars

Different wavelength light treatment conditions on different tea cultivars demonstrated a consistent reduction in PP content across various tea cultivars following exposure to wavelength-specific light treatments, though the particular wavelength inducing maximal reduction showed significant cultivar-dependent variation. Principal component analysis (PCA) of the resulting metabolic profiles revealed that cultivar-specific factors accounted for greater variance in metabolite composition than light wavelength parameters, indicating genotype exerts a more substantial influence than photic treatment on metabolic modulation. Therefore, for different tea cultivars, the most suitable treatment wavelength for reducing browning rate may be different.

Notably, KEGG analysis of these differentially expressed metabolites revealed that, regardless of whether in YF14 or YF16, the majority of differentially expressed metabolites were concentrated in metabolic pathways, specifically distributed in the Biosynthesis of other secondary metabolites, including flavone and flavonol biosynthesis.

In conclusion, the profiling of light-condition-specific metabolites and the subsequent selection of flavonoid-rich explants enable a precision-based optimization of tea plant tissue culture, allowing for the precise replication of the optimal maternal biochemical milieu.

## 4. Materials and Methods

### 4.1. Plant Material

The tea cultivars Longjing 43 (LJ43), Yunfeng 14 (YF14), and Yunfeng16 (YF16) were selected for this study. All materials were grown in November of 2012 in the experimental fields of Daqinggu, Hangzhou, China (30°20′ N, 120°07′ E). The tea plants were fertilized and watered by the same standards. In these experiments, a canopy was constructed using blackout cloth to cover the supplementary lighting area. Light tubes of different wavelengths were installed for the supplemental lighting trial. A daily supplementary lighting regimen of 7 h (from 8:00 AM to 3:00 PM) was implemented. The blackout cloth was removed outside of these hours to expose the plants to ambient natural light. Forty square meters of fresh tea leaves were exposed to irradiations of blue light (460 nm-800 lx), red light (660 nm-800 lx), near-infrared light (730 nm-800 lx), and sun light (control) for 7/17 h light/dark for 7 days, respectively. And then, shoots at the stage of one bud and two leaves were collected by manual picking on March 2024. After collection, the samples were promptly preserved in liquid nitrogen, and then freeze-dried until use.

### 4.2. Determination of Amino Acid and Polyphenols

Amino acid (AA) content was determined by the GB/T 8314-2013 [33], a national standard method, was used to determine free amino acids content of tea infusions in China, described by Ye et al. [34]. The content of each amino acid in tea powder was expressed as a percentage by mass on a dry weight basis (%). All analyses were performed with three replications for each tea sample.

The detection of tea polyphenols (PP) at the stage of one bud and two leaves was performed by UV1800 (Shimadzu, Kyoto, Japan) according to the standard protocol of GB/T 8313-2018 [35], a national standard method, used to determine of total polyphenols content of tea infusions in China [36].

### 4.3. Determination of Untargeted Metabolomics

The untargeted metabolomics were performed by LC-MS/MS analysis (Thermo UHPLC-Q Exactive system equipped with an ACQUITY BEH C18 column at Majorbio Bio-Pharm Technology Co., Ltd., Nantong, China). The mobile phases consisted of 0.1% formic acid in water:acetonitrile (2:98, *v*/*v*) (solvent A) and 0.1% formic acid in acetonitrile (solvent B). The gradient conditions are as follows: 0–0.5 min, mobile phase B was maintained at 2%; 0.5–7.5 min, mobile phase B was increased from 2% to 35%; 7.5–13 min, mobile phase B was increased from 35% to 95%; 13–14.4 min, mobile phase B was maintained at 95%; 14.4–14.5 min, mobile phase B was decreased from 95% to 2%; 14.5–16 min, mobile phase B was maintained at 2%. The flow rate was 0.40 mL/min and the column temperature was 40 °C. The injection volume was 3 µL.

### 4.4. Tissue Culture and Browning Rate Statistics

Young shoots (2–3 cm) from field-grown tea plants were surface-sterilized using 70% ethanol (1 min) followed by 2% sodium hypochlorite (10 min), then rinsed 3× with sterile distilled water. Nodal segments (1 cm) containing axillary buds were placed on MS basal medium supplemented with 1.0 mg/L BA, 0.1 mg/L NAA, 30 g/L sucrose, and 7 g/L agar (pH 5.8). The incubation was performed in the dark for 72 h at 25 ± 1 °C, followed by a photoperiod of 16 h (30 µmol/m^2^/s cool white fluorescent lamps) for 7 days. Browning rate (%) = (Number of Browning Explants/Total Number of Inoculated Explants) × 100%.

### 4.5. Data Analysis

The pretreatment of LC/MS raw data was performed by Progenesis QI (Waters Corporation, Milford, MA, USA) software, and a three-dimensional data matrix in CSV format was exported. At the same time, the metabolites were identified by searching a self-built plant-specific metabolite database (MJDBPM).

The data were analyzed through free online platform majorbio cloud platform (https://www.majorbio.com/) [37]. Metabolic features that were detected at at least 80% in any set of samples were retained. Meanwhile, variables with relative standard deviation (RSD) > 30% of QC samples were removed, and log10 processing was performed to obtain the final data matrix for subsequent analysis.

T Data for AA, PP were analyzed using SPSS software (version 25.0, IBM SPSS Statistics, Chicago, IL, USA). After testing for a normal distribution, a multiple test was applied. Results were considered significant when *p* < 0.01, and the graphs drawn using the IBM SPSS Statistics 25.0 and Python 3.8.12 [3].

## 5. Conclusions

This study demonstrates that wavelength-specific light treatments can induce reversible modifications in the physicochemical characteristics of tea leaves, effectively reducing the accumulation of flavonoid compounds, including polyphenols, in plant tissues. Notably, tissues subjected to optimized wavelength conditions exhibit superior performance as explant sources for in vitro culture systems, demonstrating significantly lower browning rates. Comparative analysis of 460 nm, 660 nm, and 730 nm irradiation treatments revealed consistent suppression of polyphenol biosynthesis across all examined cultivars. However, the wavelength eliciting maximal reduction showed significant cultivar-dependent variation, indicating genotype-specific photoresponsive regulation of secondary metabolism.

## Figures and Tables

**Table 2:** Twenty-four compounds that exhibited a significant decrease relative to the control group.

Metabolite	Decrease Ratio Compared to the Control Group (%)		
	B_460	R_660	R_730
Gingerglycolipid A	−54%	−61%	−43%
6Z-8-Hydroxygeraniol 8-O-glucoside	−44%	−59%	−47%
Kaempferol-3-O-glucoside	−63%	−64%	−42%
(2E,4E)-2,4-Hexadienoic acid	−45%	−43%	−38%
4-(3-hydroxybutyl)-2-methoxyphenol	−36%	−41%	−37%
Dulxanthone A	−62%	−76%	−85%
Podecdysone B	−45%	−65%	−46%
Adenosine 3′-monophosphate	−62%	−67%	−28%
Hyperoside	−56%	−77%	−76%
5-Hydroxy-p-mentha-6,8-dien-2-one	−48%	−47%	−73%
Liquiritigenin 4′-[3-acetylapiosyl-(1->2)-glucoside]	−72%	−62%	−58%
Myricetin 7-(6″-galloylglucoside)	−83%	−87%	−63%
(−)-Epigallocatechin 3-cinnamate	−85%	−84%	−69%
Guanidylic acid (guanosine monophosphate)	−66%	−63%	−47%
Chondroitin 6′-sulfate	−66%	−70%	−50%
(7′R,8′R)-4,7′-Epoxy-3′-methoxy-4′,5,9,9′-lignanetetrol 9′-glucoside	−79%	−72%	−56%
Eriodictyol	−78%	−80%	−70%
Phaseollinisoflavan	−74%	−70%	−60%
(+/−)-Naringenin	−75%	−74%	−64%
Gamma-delta-Dioxovaleric acid	−83%	−84%	−82%
Eriodictyol-7-O-glucoside	−78%	−88%	−86%
Naringenin-7-O-glucoside	−69%	−79%	−68%
Succinylacetone	−60%	−78%	−63%
Ethyl beta-D-glucopyranoside	−92%	−79%	−90%

## Data Availability

The data of this article can be found in the “Non-targeted metabolomics data of tea plants under different light conditions”, Mendeley Data, V1, doi: 10.17632/kcy2btkf3p.1.

## Abbreviations

The following abbreviations are used in this manuscript:

AC	Activated carbon
PVP	polyvinyl pyrrolidone (PVP)
LJ43	Longjing 43
YF14	Yunfeng14
YF16	Yunfeng16
AA	Amino acid
PP	Polyphenols
PP/AA	Phenol-Ammonia Ratio
